# Pelvic autonomic dysfunction is common in patients with pure autonomic failure

**DOI:** 10.1111/ene.16486

**Published:** 2024-09-30

**Authors:** E. Vichayanrat, C. Hentzen, S. Simeoni, M. Pakzad, V. Iodice, Jalesh N. Panicker

**Affiliations:** ^1^ Department of Uro‐Neurology National Hospital for Neurology and Neurosurgery London UK; ^2^ Autonomic Unit National Hospital for Neurology and Neurosurgery London UK; ^3^ UCL Queen Square Institute of Neurology, Faculty of Brain Sciences University College London London UK; ^4^ Sorbonne Université GRC 01, GREEN Groupe de Recherche Clinique en Neuro‐Urologie, AP‐HP Paris France

**Keywords:** bowel dysfunction, lower urinary tract dysfunction, nocturnal polyuria, pelvic autonomic dysfunction, pure autonomic failure

## Abstract

**Background and Purpose:**

Pure autonomic failure (PAF) presents primarily as cardiovascular autonomic failure and may phenoconvert to other neurodegenerative disorders. However, the involvement of other autonomic functions has been poorly evaluated. This study aims to characterize genitourinary and bowel dysfunction and explore their relationship with cardiovascular autonomic dysfunction.

**Methods:**

Pure autonomic failure patients underwent cardiovascular autonomic testing and an assessment of pelvic autonomic dysfunction using urinary, sexual symptoms questionnaires and a bladder diary. Demographic, clinical features and related medical comorbidities were assessed.

**Results:**

Twenty‐five patients (10 males) with PAF were included (mean age 71 ± 8 years; disease duration 13 ± 8 years). 96% (24/25) reported lower urinary tract symptoms, of which overactive bladder symptoms were most commonly reported (*n* = 23; 92%; median overactive subscore 8, interquartile range [IQR] 3–11), followed by voiding difficulties (*n* = 19; 76%; median low stream subscore 2, IQR 1–3) using the Urinary Symptom Profile; however, only four (16%) required clean intermittent self‐catheterization. Sexual dysfunction was common (*n* = 21; 84%) using the Arizona Sexual Experience Scale. Mild faecal incontinence and constipation were reported. 86% (19/22) had nocturnal polyuria (NP) and the median NP index was 47% (IQR 38%–51%; normal range <33%). 77% (10/13) had voiding dysfunction and 31% (4/13) had post‐void residual urine >100 mL. There were no significant correlations between the need for catheterization and the degree of NP with age, disease duration and cardiovascular autonomic parameters (*p* > 0.05).

**Conclusions:**

Nocturnal polyuria, genitourinary and bowel symptoms are commonly seen in PAF. The pathophysiology of NP in PAF is most likely multifactorial and may occur independent of cardiovascular autonomic failure.

## INTRODUCTION

Pure autonomic failure (PAF) is a sporadic neurodegenerative disorder characterized by accumulation of misfolded alpha‐synuclein in the form of neuronal cytoplasmic inclusions called Lewy bodies, which phenotypically presents as autonomic failure without neurological symptoms and signs [1]. Patients with PAF usually present with orthostatic intolerance symptoms such as dizziness, visual disturbances, light‐headedness, loss of consciousness, temporary cognitive impairment, neck muscle pain (‘coat hanger’ pain) or collapse as a result of cardiovascular autonomic failure [2]. However, other autonomic symptoms such as nocturia, bowel and sexual problems are also seen. A recent natural history study of patients with PAF demonstrated that 50% reported lower urinary tract (LUT) symptoms and up to 65% of men reported erectile dysfunction within 5 years of disease onset [3]. A substantial proportion of patients, ranging between 12% and 34%, evolve into other forms of synucleinopathy such as multiple system atrophy (MSA), Parkinson's disease (PD) and dementia with Lewy bodies [4, 5, 6, 7], and severe bladder dysfunction, particularly urinary retention, has been shown to be a predictor of phenoconversion to MSA [7]. Gastrointestinal symptoms such as constipation have also been reported in patients with PAF and may even be a presenting feature [8].

Despite the high prevalence of genitourinary and bowel symptoms in PAF, these features are often overlooked and have never been systematically investigated. Furthermore, the prevalence of these symptoms and their association with other autonomic symptoms in PAF remains unclear. The aim of this study therefore was to characterize pelvic autonomic dysfunction including LUT, bowel and sexual symptoms in PAF patients, and to explore their relationship with cardiovascular autonomic dysfunction.

## METHODS

In this cross‐sectional study, all patients fulfilling the diagnostic criteria of PAF [1] and followed up in the Autonomic Unit at a tertiary level academic teaching hospital for at least 5 years without developing other neurological features between November 2019 and October 2021 were approached this study. The study was registered and approved by the Queen Square Quality and Safety Team (reference 79‐202,021‐CA) as a service evaluation and the study protocol and data collection were conducted in accordance with the ethical standards as per the Declaration of Helsinki; written informed consent was obtained from all patients.

### Clinical history

Case notes were reviewed and history and relevant information including age, gender, duration of disease (time from diagnosis to testing date), onset of urinary, gastrointestinal and sexual symptoms, medications, obstetric history, and other medical comorbidities such as congestive heart failure, sleep apnoea or previous history of lumbar spondylosis or surgeries including any urological or gynaecological procedures were noted. None of the PAF patients had a history of diabetes mellitus, peripheral neuropathy or other illnesses that could affect autonomic function.

Participants completed a self‐report form enquiring whether they experienced urinary, bowel or sexual symptoms and duration of symptoms. Standardized validated questionnaires evaluating LUT, sexual and gastrointestinal symptoms were then administered.

- The Urinary Symptom Profile (USP) questionnaire is a 13‐item questionnaire exploring three domains of LUT symptoms: overactive bladder symptoms (score 0–21), low stream (voiding) (score 0–9) and stress urinary incontinence (score 0–9), higher scores indicating greater symptoms [9].
- The eight‐item short form (SF) Qualiveen assesses the impact that urinary symptoms have on quality of life [10]. This questionnaire assesses how different aspects of patients' lives are affected and limited by their urinary symptoms.
- The Arizona Sexual Experiences Scale (ASEX) explores different domains of sexual functions and the total score ranges between 5 and 30. Higher scores indicate greater symptoms, and sexual dysfunction was defined as a total score of 19 or greater, or when one of the single items scored 5 or 6, or when any three items had a score of 4 or higher [11].
- The Wexner Incontinence Score evaluates bowel incontinence and the questionnaire consists of five items related to incontinence and lifestyle alteration. The severity of each item was calculated on a scale ranging from 0 to 20, higher scores indicating greater symptoms: 1–8 minor faecal incontinence, 9–14 average faecal incontinence and 15–20 complete faecal incontinence [12].
- The eight‐item Constipation Scoring System (CSS) assesses the severity of constipation and a higher overall CSS score indicates more severe constipation: 0–10 mild, 10–20 moderate and 21–30 severe constipation [13].

Patients were asked to complete a standard 3‐day bladder diary at home measuring their daily fluid intake and individual urine output volumes and recording each time they voided. Bedtime, for sleep, wake‐up time, night‐time/daytime frequency and mean night‐time/daytime voided volume were also recorded. To evaluate nocturnal urine production, the nocturnal polyuria index (NPi) was calculated and nocturnal polyuria (NP) was defined as a ratio of nocturnal urine volume/24‐h urine volume greater than 33% in the elderly (age >65 years) [14].

### Urodynamic studies

Patients reporting significant LUT symptoms underwent urodynamic studies. Urinary flow when voiding with the sensation of a full bladder was assessed non‐invasively by uroflowmetry (Albany Medical SmartFlow) and the post‐void residual volume (PVR) was measured using a portable bladder ultrasound scanner (Bardscan Realtime). A subset of patients underwent invasive multichannel urodynamic studies as part of their clinical assessments. These include filling cystometry (medium fill at 50 mL/min) and pressure–flow studies (Medical Measurement Systems, Dover, NH, USA).

### Cardiovascular autonomic screening tests and 24‐h ambulatory blood pressure and heart rate monitoring

Cardiovascular autonomic function tests and 24‐h ambulatory blood pressure monitoring were performed following standard protocols at the Autonomic Unit [15] and tests were conducted within a 6‐month period of each other. Data from autonomic function tests that included 10‐min head‐up tilt test, deep breathing and Valsalva manoeuvre were extracted. The head‐up tilt test was performed at 60° for 10 min. Deep breathing was performed at a rate of 6 breaths per minute for 1 min and the Valsalva manoeuvre was performed by blowing against a fixed resistance at 40 mmHg for 15 s in the supine position.

Continuous beat‐to‐beat blood pressure was measured using digital photoplethysmography (Finapres, TNO‐TPD, Biomedical Instrumentation), as well as cardiac activity with electrocardiogram monitoring. The blood pressure responses were derived from the Valsalva manoeuvre using on‐line data acquisition software (Chart version 7.1, ADInstruments, Oxford, UK).

Patients fitted with 24‐h ambulatory blood pressure monitoring (model 90,207, Spacelabs™ Medical, Redmond, WA, USA) were asked to record their symptoms in a diary during the day (e.g., dizziness, light‐headedness, blurred vision etc.) as well as their position and activity (lying down, sitting, standing, walking and exercising) simultaneously. The timings of bedtime for sleep and wake‐up time were also recorded in order to determine the period of night‐time and daytime. Blood pressure and heart rate were recorded every 20 min during the day and every 60 min during the night. The average blood pressure and heart rate were calculated for daytime, night‐time and the entire 24‐h period. Symptoms (if experienced by patients) were also noted in the diary. Blood pressure and heart rate variabilities were derived from the standard deviations of the means of the specific period of time (24 h, daytime and night‐time).

According to diurnal blood pressure variability, patients were classified into three groups: dipper (blood pressure fall during night‐time >10% compared to daytime), non‐dipper (blood pressure fall during night‐time <10% compared to daytime) and reverse nocturnal blood pressure (blood pressure higher during night‐time than daytime). Patients with an average daytime systolic blood pressure >140 mmHg or diastolic blood pressure >90 mmHg were defined as daytime hypertensives and those with night‐time systolic blood pressure >125 mmHg or diastolic blood pressure >75 mmHg as night‐time hypertensives [16].

Orthostatic hypotension is defined as a fall in systolic blood pressure of >20 mmHg or diastolic blood pressure of >10 mmHg on standing or during head‐up tilt [17].

## STATISTICAL METHODS

Descriptive analysis was conducted to calculate the mean, range and standard deviation (SD) of the patient demographics, clinical characteristics and scores from the questionnaires. Mann–Whitney *U* tests were used to compare continuous variables between the two groups for non‐normally distributed data and unpaired *t* tests for normally distributed data. Chi‐squared analyses were used for analysis of categorical variables. The relationships between cardiovascular autonomic function indices and other factors, such as disease duration and disease severity (continuous variables), were assessed by correlation analysis (Pearson's correlation for normally distributed data and Spearman's correlation for non‐normally distributed data). Statistical analyses were carried out using Stata 11.0 (Stata Corporation, College Station, TX, USA). All tests were two‐sided and a *p* value of ≤0.05 was considered significant.

## RESULTS

Twenty‐five PAF patients (10 males) were included. All patients developed LUT symptoms after their orthostatic intolerance symptom. Other associated medical conditions such as enlarged prostate (five patients), hysterectomy (four patients) and prolapse and sleep apnoea (*n* = 2 each) were reported. The other patients' comorbidities and medications are presented in Table S2.

Demographic data and questionnaire scores are presented in Table 1. LUT symptoms were reported in 96% (24/25) of patients using the USP questionnaire and overactive bladder and voiding symptoms were reported in 92% (23/25) and 76% (19/25), respectively. Four (16%) patients required intermittent self‐catheterization. Sexual dysfunction was present in 84% (21/25) using the ASEX questionnaire. The CSS questionnaire median score was 7 (interquartile range [IQR] 4–13) and 79% (19/24) of patients reported mild constipation. The median Wexner incontinence median score was 7 (IQR 3–9) and more than half of patients (54%; 13/24) reported minor faecal incontinence and none had severe constipation (Table 1 and Figure S1). Other scores and bladder diary findings are presented in Table 1.

**TABLE 1:** Patient characteristics and reported pelvic autonomic symptoms of 25 patients with pure autonomic failure.

Variable	*N*
Gender (M:F)	10:15
Age, years	71 ± 8
Disease duration, years	13 ± 8
Interval between onset of orthostatic intolerance and LUT symptoms, median (IQR), years	4 (2–9)
Renal functions (*n* = 19)	
Mean creatinine, μmol/L (normal range 66–112 μmol/L)	96 ± 23
Mean eGFR, mL/min	63 ± 17
Chronic Kidney Disease staging according to eGFR (<60 mL/min per 1.73 m^2^)	
Stage 1 Normal (eGFR > 90 mL/min)	21% (4/19)
Stage 2 (eGFR 60–89 mL/min)	32% (6/19)
Stage 3 (eGFR 30–59 mL/min)	47% (9/19)
Bladder diary findings (*n* = 22)	
Night‐time frequency, *n*	2.5 (1.18)
Daytime frequency, *n*	6.6 (2.78)
Mean night‐time voided volume, mL	913 (496)
Mean daytime voided volume, mL	1137 (591)
Nocturnal polyuria index, median (IQR)	47% (IQR 38%–51%)
Fluid intake, mL	2041 (603)
USP, median (IQR)	
Overactive subscore (0–21)	8 (3–11)
Low stream subscore (0–9)	2 (1–3)
Stress incontinence subscore (0–9)	1 (0–4)
Constipation Scoring System (0–30)	
Mild (0–15)	79%
Severe (>15)	21%
Wexner Incontinence Score (0–20)	
No incontinence (score 0)	13%
Minor faecal incontinence (score 1–8)	54%
Average faecal incontinence (score 9–14)	25%
Complete faecal incontinence (score 15–20)	8%
ASEX score, median (IQR)	17 (14–19)
SF‐Qualiveen	1.6 ± 1.1
Bother with limitations	1.6 ± 1.2
Fears	1.6 ± 1.2
Feeling	1.5 ± 1.3
Frequency of limitations	1.6 ± 1.2

*Note*: Values are mean ± SD unless stated.

Abbreviations: ASEX, Arizona Sexual Experiences Scale; eGFR, estimated glomerular filtration rate; IQR, interquartile range; LUT, lower urinary tract; SF‐Qualiveen, short form Qualiveen score; USP, Urinary Symptom Profile questionnaire.

All patients had confirmed cardiovascular autonomic failure with abnormal heart rate responses to respiratory stimuli and orthostatic hypotension on head‐up tilt. Supine hypertension and reversed circadian blood pressure rhythm was present in 81% (17/21) and 57% (12/21), respectively, on 24‐h ambulatory blood pressure monitoring (Table S1). Of these, eight (36%) PAF patients voided twice at night, followed by three times in six patients (27%), once at night in four patients (18%) and four and five times in two patients each. Amongst 13 patients who underwent uroflowmetry, 77% (10/13) demonstrated evidence for voiding dysfunction as per uroflowmetry and only three patients had normal flow. 31% (4/13) required intermittent self‐catheterization as the PVRs were more than 100 mL consistently. Six patients underwent cystometrography, which showed detrusor overactivity (DO; *n* = 2) and detrusor underactivity (DU; *n* = 4) according to the bladder contractility index and only one patient had a combination of DO and DU. The other cystometrogram findings are presented in Table 2.

**TABLE 2:** Cystometrogram findings in six patients with pure autonomic failure.

Gender/age at testing (years)	Disease duration (years)	First sensation (mL)	Strong desire (mL)	Compliance	DO (no = 0, yes = 1)	Maximum cystometric capacity (mL)	*Q*_max_ (mL/s)	*P*_det_ *Q*_max_ (cmH_2_O)	PVR (mL)	Voided volume (mL)	BCI^a^
Female/79	11	266	Not reached	Normal	0	554	9	31	6	548	76 (weak)
Male/66	11	254	396	Normal	0	520	9	45	97	291	90 (weak)
Female/78	20	150	260	Normal	0	440	15	25	0	400	100 (normal)
Male/76	6	140	200	Normal	1	200	17	55	42	160	140 (strong)
Female/62	7	116	141	Normal	1	176	10	18	2	149	68 (weak)
Female/57	6	260	565	Normal	0	600	8	10	100	470	50 (weak)

Abbreviations: BCI, bladder contractility; DO, detrusor overactivity; *P*_det_, detrusor pressure; PVR, post‐void residual urine; *Q*_max_, maximum flow rate.

^a^ BCI is the bladder contractility index based on detrusor pressure at maximum flow rate (*P*_det_ at *Q*_max_) and maximum flow rate (*Q*_max_); BCI > 150 = strong contractility, BCI 100–150 = normal contractility and BCI < 100 = weak contractility.

A bladder diary is difficult to maintain. However, 22/25 (88%) patients adequately completed the bladder diary. Of the 22 patients who completed the bladder diary, 19 (86%) had NP with a mean NPi of 47% (IQR 38%–51%). Correlations between NP and voiding dysfunction with demographic and cardiovascular findings are presented in Table 3. There were no significant correlations between NP or voiding dysfunction with age, disease duration and cardiovascular parameters (orthostatic blood pressure drop, supine hypertension, heart rate responses to deep breathing, Valsalva ratio) and circadian blood pressure parameters on 24‐h ambulatory blood pressure monitoring between There were no significant correlations in age, disease duration, and cardiovascular parameters (orthostatic blood pressure drop, supine hypertension, heart rate responses to deep breathing, Valsalva ratio), or circadian blood pressure parameters on 24‐hour ambulatory blood pressure monitoring, neither between PAF patients with and without NP, nor between those with and without voiding dysfunction (p > 0.05). However, the nocturnal blood pressure was significantly higher in PAF patients without urinary catheterization than those with urinary catheterization (mean percentage of nocturnal systolicblood pressure fall; 8% ± 9% vs. −10% ± 14%; *p* ≤ 0.02) (Table 3).

**TABLE 3:** The distribution of nocturnal polyuria and voiding dysfunction across demographic and cardiovascular findings in 22 patients with pure autonomic failure.

Variable	(1) Nocturnal polyuria		(2) Voiding dysfunction	
	NPi < 33%^a^	NPi > 33%^a^	Catheterizing	Not catheterizing
Number of patients	3	19	3	19
Age, median (IQR), years	77 (62–78)	68 (64–77)	60 (57–78)	71 (65–77)
Disease duration, median (IQR), years	12 (9–12)	11 (7–15)	7 (6–12)	11 (9–15)
Bladder diary findings				
Night‐time frequency, *n*	1.8 (1.35)	2.6 (1.16)	2.2 (2.12)	2.5 (1.06)
Daytime frequency, *n*	7 (3.79)	6.6 (2.72)	4 (0.88)	6.3 (2.7)
Mean night‐time voided volume, mL	695.6 (383.3)	947.5 (511.3)	1252.2 (887.4)	859.6 (410.9)
Mean daytime voided volume, mL	1976 (836)	1005 (441)*	1461 (877)	1086 (550)
Fluid intake, mL	2116 (782)	2027 (595)	1619 (321)	2121 (617)
Creatinine, μmol/L	122 ± 13.75	88.78 ± 21.43*	101 ± 33.94	93.8 ± 23.65
eGFR, mL/min	53.3 ± 5.86	68.36 ± 16.67	70.5 ± 27.58	65.07 ± 4.04
CV autonomic testing				
Mean supine SBP	164 ± 7	167 ± 27	148 ± 17	171 ± 26
Orthostatic ∆SBP	−82 ± 29	−82 ± 37	−56 ± 37	−89 ± 34
HR responses to deep breathing	1 ± 1	4 ± 4	3 ± 2	4 ± 4
Valsalva ratio	1.2 ± 0.2	1.2 ± 0.2	1.2 ± 0.1	1.2 ± 0.2
24‐h ABPM				
Mean SBP daytime (mmHg)	136 ± 15	124 ± 20	122 ± 22	127 ± 19
Mean SBP night‐time (mmHg)	135 ± 11	134 ± 23	114 ± 22	138 ± 20*
Mean nocturnal SBP fall (%)	0 ± 3	−9 ± 16	8 ± 9	−10 ± 14*

*Note*: Values are mean (SD), if not indicated.

Abbreviations: ∆SBP, change in systolic blood pressure; 24‐h ABPM, 24‐h ambulatory blood pressure monitoring; CV, cardiovascular; eGFR, estimated glomerular filtration rate; HR, heart rate; IQR, interquartile range; NPi, nocturnal polyuria index; SBP, systolic blood pressure.

^a^ NPi is the nocturnal polyuria index; if 33%, it suggests nocturnal polyuria.

* *p* < 0.05.

## DISCUSSION

This study aimed to characterize LUT, bowel and sexual symptoms in PAF patients and explore the relationship between these autonomic symptoms with cardiovascular autonomic dysfunction. Our study demonstrates that standardized self‐reported questionnaires and a bladder diary are useful tools to screen for symptoms of pelvic organ dysfunction in PAF patients. Both overactive bladder (92%) and voiding symptoms (76%) were the most commonly reported LUT symptoms in our study. Amongst patients with voiding symptoms, at least 16% (4/25) required catheterization. These findings are consistent with the subset of our patients who underwent urodynamics studies and demonstrated DO, sensory urgency and DU. Both constipation and faecal incontinence were also commonly reported and were of mild severity.

Pure autonomic failure is considered a predominantly post‐ganglionic autonomic disorder and this is supported by the finding of alpha‐synuclein aggregation as neuronal cytoplasmic inclusions in the intermediolateral column of the spinal cord with accompanying neuronal loss, Lewy bodies in sympathetic ganglia, enteric neuron bladder wall [18] and skin sympathetic nerves [19]. The mechanism of LUT dysfunction in patients with PAF is unclear. However, the neuropathological findings are, at least in part, likely to be responsible for genitourinary and bowel symptoms in patients with PAF. Nevertheless, DO on the urodynamics finding in our patients and detrusor sphincter dyssynergia in some PAF patients in an earlier reported case series [20] suggest possible involvement of central micturition control in these patients. The storage and voiding LUT symptoms are common in our cohort of PAF and our findings suggest that these may be more common than those with PD and dementia with Lewy bodies but less common than MSA [21].

Nocturia, the complaint of having to wake up at night one or more times to void [22], is one of the most common LUT symptoms reported in patients with PD, another form of alpha‐synucleinopathy [23]. The prevalence of nocturia in PD varies considerably, ranging from 53% to 78% [24, 25], and has a significant impact on patients' quality of life as it is associated with sleep disturbances, greater risk for falls and hip fractures, and higher mortality in older individuals [26, 27, 28, 29, 30].

A previous study showed that NP is also prevalent in PD, another form of synucleinopathy [23, 31, 32]. However, our findings suggest that this may be more prevalent in PAF compared to PD [23]. The exact mechanism of NP remains unclear and there are possible different mechanisms that could result in NP. Abnormal salt and water homeostasis regulation plays an important role in pathogenesis of NP [33]. In addition, NP is also a common manifestation in chronic kidney disease and there are several potential mechanisms that account for this. These include the inability to concentrate urine due to reduced medullary interstitial function and diminished responsiveness of the collecting duct to arginine vasopressin (AVP), which leads to water diuresis, in conjunction with third‐space fluid sequestration [34]. The urinary bladder urothelium has recently been proposed to be involved in sensory and transducer function via a network of functional receptors and ion channels that interacts with bladder mucosa and other components in the bladder wall. Functional components of the urinary bladder urothelium also play an important role in AVP‐mediated water homeostatic, which are contributing factors to the aetiology of NP [35]. It is unclear when NP occurs in patients with PAF. This warrants further investigation whether there are other factors that could explain the difference in prevalence of NP amongst different forms of synucleinopathies.

Orthostatic hypotension as part of cardiovascular autonomic failure and abnormal circadian blood pressure rhythm are characteristic features of patients with PAF. These features are also known to be factors associated with nocturia [36] and NP in PD [37].To our knowledge, this is the first study that investigated the relationship between NP and cardiovascular autonomic parameters including circadian blood pressure rhythms on 24‐h ambulatory blood pressure monitoring. Surprisingly, no clear association between NP and any of the cardiovascular autonomic parameters, abnormal circadian blood pressure rhythm on 24‐h ambulatory blood pressure monitoring or disease duration was found. Similarly, voiding dysfunction that arises from a loss of parasympathetic innervation to the detrusor was evaluated with other CV parameters and no association was seen. PAF patients without significant voiding dysfunction had higher degrees of reversed nocturnal blood pressure changes compared to those with voiding dysfunction requiring urinary catheterization. The reason for this is unclear; however, it is possible that the mean nocturnal blood pressure fall is more marked when the patients get up at night for catheterization. There was no difference in the night‐time frequency between the two groups.

Our study demonstrated that renal impairment was prevalent in patients with PAF, consistent with the earlier study [38]. Supine hypertension was thought to be a contributing factor for chronic kidney disease in PAF [38]. PAF patients with NP have significantly lower levels of serum creatinine and the higher creatinine levels in patients with lower NPi may reflect a more aggressive disease progression in these patients. Nonetheless, the cause of renal impairment in PAF has not been explored sufficiently and warrants further investigation. Urinary symptoms had an impact on PAF patients' quality of life as demonstrated by SF‐Qualiveen scores. These findings are in line with the previous study showing that nocturia with at least two voids per night has an impact on health‐related quality of life [39].

Only a proportion of patients underwent urodynamics and therefore it was not possible to conclude the patterns of LUT dysfunctions underpinning symptoms in this cohort. Cystometrogram is a relatively invasive test and currently not recommended in all patients and hence was not performed in everyone [40]. The interpretation of results was limited by the lack of an age‐matched cohort without PAF for comparison and therefore it would not be possible to factor in age‐related LUT symptoms and NP in elderly people. However, the previous data from epidemiological studies in non‐neurological patients suggest that NP is also prevalent in this age range [41, 42]. Whether NP is simply an age‐related change or related to underlying PAF needs to be further investigated having an age‐matched non‐PAF control. Furthermore, the size of the subgroups compared is relatively small (e.g., NPi <33% vs. NPi >33%, catheterizing vs. not catheterizing) and it is difficult to draw a conclusion from our findings. Causes for NP were assessed by a chart review at our subspecialty clinics and a formal multidisciplinary assessment was lacking. Therefore, the prevalence of comorbidities such as heart failure and sleep apnoea which can be associated with NP may have been underestimated. Lastly the cohort is likely to represent a more severe presentation of PAF reporting at a tertiary hospital. A prospective design in a larger cohort of PAF including patients presenting at an earlier stage of PAF would provide more accurate outcomes in determining the natural history of NP in these patients. The results of Dopamine transporter scan and Meta‐iodobenzylguanidine scans, sudomotor assessment, skin biopsy and cerebrospinal fluid analysis were not used to phenotype the cohort. The role of these investigations in predicting phenoconversion to other synucleinopathies is still being researched and beyond the remit of this paper.

To our knowledge, this study represents the first characterization of pelvic autonomic symptoms in patients with PAF. The strength of this study is the detailed nature of the assessments as specialists' input from both uro‐neurology and autonomic specialists was involved in designing the study and interpreting results. All patients were followed up for longer than 5 years to ensure that they were not evolving to develop other forms of alpha‐synucleinopathies such as MSA or other parkinsonian disorders.

A longitudinal study that phenotypes the LUT dysfunction and NP is required to better understand the mechanisms for NP PAF and the progression of pelvic autonomic dysfunction over time. Screening for and treating pelvic autonomic symptoms in patients with PAF are strongly recommended given their impact on quality of life. Desmopressin has been shown to be an effective treatment for managing NP in patients with synucleinopathy, in particular PD. Further studies are required to establish whether or not this works in patients with PAF [43].

In summary, genitourinary and gastrointestinal symptoms are commonly reported in patients with PAF. Storage urinary symptoms and NP are present in >85% of patients and voiding dysfunction is present in >75% of patients and 16% of PAF patients require urinary catheterization. The pathophysiology of NP in PAF is likely to be multifactorial in view of the lack of correlation with cardiovascular autonomic failure/abnormal circadian blood pressure rhythm. Furthermore, voiding dysfunction does not appear to be correlated with disease duration and severity of cardiovascular dysfunction in PAF. The standardized questionnaires and bladder diary are useful in detecting genitourinary and bowel symptoms and therefore should be used as part of uro‐neurological assessments in patients with PAF.

## AUTHOR CONTRIBUTIONS

**E. Vichayanrat:** Conceptualization; methodology; software; data curation; investigation; writing – original draft; writing – review and editing; formal analysis. **C. Hentzen:** Conceptualization; data curation; investigation; methodology; writing – review and editing. **S. Simeoni:** Writing – review and editing; supervision. **M. Pakzad:** Supervision; writing – review and editing. **V. Iodice:** Supervision; writing – review and editing; conceptualization. **Jalesh N. Panicker:** Conceptualization; methodology; supervision; resources; project administration; writing – review and editing; validation; funding acquisition.

## CONFLICT OF INTEREST STATEMENT

All authors declare no competing interest.

## Supporting information


Figure S1.

Table S1.

Table S2.

## Data Availability

The data that support the findings of this study are available from the corresponding author upon reasonable request.
