# Supplementary figures and images for: Pelvic autonomic dysfunction is common in patients with pure autonomic failure
Source: Eur J Neurol. 2024 Sep 30;31(12):e16486. doi: 10.1111/ene.16486 (PMC11555151; doi:10.1111/ene.16486)

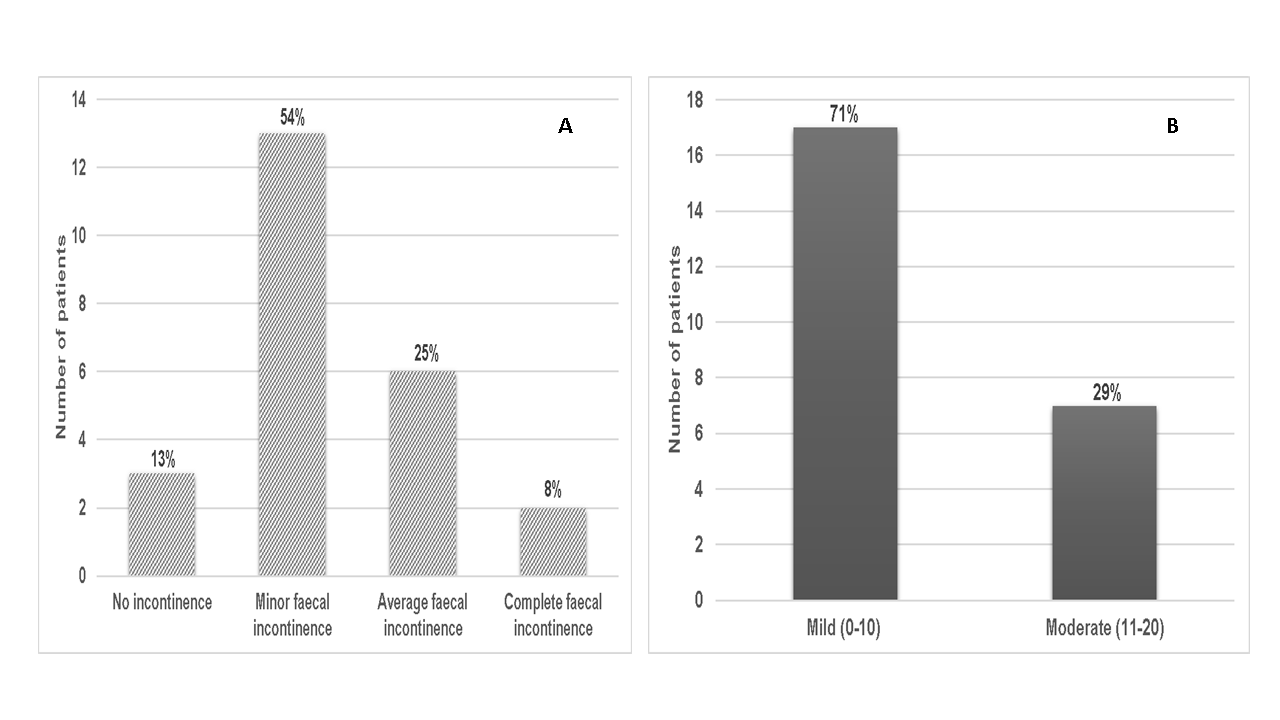

Supplement: Supplementary file 1 — Figure S1. [file ENE-31-e16486-s002.tif]
