# Supplementary material for: Pelvic autonomic dysfunction is common in patients with pure autonomic failure
Source: Eur J Neurol. 2024 Sep 30;31(12):e16486. doi: 10.1111/ene.16486 (PMC11555151; doi:10.1111/ene.16486)
Supplement: Supplementary file 2 — Table S1. [file ENE-31-e16486-s003.docx]

**Supplementary Table 1**

**Cardiovascular autonomic function tests and 24-hour ABPM profiles in 22 patients with PAF**

| **Variable** | **PAF (n=22)** |
| --- | --- |
| **Gender (M:F)** | 10:12 |
| **Age, years** | 70+7 |
| **Disease duration, years** | 12+5 |
| **Autonomic function tests**  ***HUT***  -Supine SBP  -Tilt SBP  -Orthostatic ∆ SBP  ***HR_DB_***  ***Valsalva ratio*** | 166+25  82+28  84+35  1+0.2  1.22+0.16 |
| **Patients with supine hypertension** | 82% (18/22) |
| **24-hour ABPM profiles**  ***Daytime***  - Mean SBP  - Mean DBP  - Mean HR  ***Night-time***  - Mean SBP  - Mean DBP  - Mean HR | 126+19  76+11  69+9  134+21  78+10  64+11 |
| ***BP circadian rhythm, % (n)***  -Patients with normal BP circadian rhythm, % (n)  -Patients with absent BP circadian rhythm, % (n)  -Patients with reversed BP circadian rhythm, % (n) | 9% (2/22)  32% (7/22)  59% (13/22) |
| Values are mean+SD unless stated, HUT, Head-up tilt; SBP, systolic blood pressure; DBP, diastolic blood pressure; Orthostatic ∆ SBP, systolic blood pressure changes during HUT; HR***_DB,_*** Heart rate responses during deep breathing; 24-hour ABPM, 24-hour ambulatory blood pressure monitoring | |
