# Supplementary material for: Pelvic autonomic dysfunction is common in patients with pure autonomic failure
Source: Eur J Neurol. 2024 Sep 30;31(12):e16486. doi: 10.1111/ene.16486 (PMC11555151; doi:10.1111/ene.16486)
Supplement: Supplementary file 3 — Table S2. [file ENE-31-e16486-s001.docx]

**Supplementary Table 2**

**Comorbidities and medications in 25 patients with Pure Autonomic Failure**

| **Gender/ Age at testing (years)** | **Comorbidities** | | | | **Medications** | | | | | | |
| --- | --- | --- | --- | --- | --- | --- | --- | --- | --- | --- | --- |
|  | **Benign prostate enlargement** | **Pelvic organ surgery** | **Obstructive sleep apnea** | **Previous stroke** | **Anti-muscarinic agent** | **Mirabegron** | **Fludrocortisone** | **Midodrine** | **Calcium channel blocker** | **Nitroglycerin** | **ACE inhibitor** |
| Female/78 | - | - | + | - | - | - | - | - | - | - | - |
| Male/60 | - | - | + | - | - | - | - | - | - | - | - |
| Female/72 | - | - | - | - | - | - | + | - | - | - | - |
| Female/82 | - | - | - | + | - | - | - | - | - | - | - |
| Male/77 | - | - | - | - | - | - | - | - | - | - | - |
| Female/68 | - | + | - | - | - | - | - | + | + | + | - |
| Female/77 | - | + | - | - | - | - | + | - | - | - | - |
| Male/66 | - | - | - | - | - | - | + | + | - | - | - |
| Female/64 | - | - | - | - | - | - | + | + | - | - | - |
| Female/79 | - | - | - | - | - | - | - | - | - | - | - |
| Female/59 | - | - | - | - | - | - | + | + | - | - | - |
| Female/65 | - | - | - | - | - | - | - | + | - | - | + |
| Male/68 | - | - | - | - | - | - | + | + | - | - | - |
| Male/65 | - | - | - | - | - | - | - | + | - | - | - |
| Female/74 | - | + | - | - | - | - | - | - | - | - | - |
| Female/74 | - | - | - | - | - | - | - | + | - | - | - |
| Female/89 | - | - | - | - | - | - | + | - | - | - | - |
| Male/71 | + | - | - | - | - | + | + | + | - | - | - |
| Male/80 | + | - | - | - | - | - | - | - | - | - | - |
| Male/80 | + | - | - | - | - | - | - | - | + | - | + |
| Female/75 | - | - | - | - | - | - | - | - | - | - | - |
| Female/63 | - | + | - | - | + | - | - | + | - | - | - |
| Male/78 | + | - | - | - | - | - | + | + | - | - | - |
| Female/57 | - | - | - | - | - | - | + | + | - | - | - |
| Male/62 | + | - | - | - | - | - | - | - | - | - | - |
| *+ = present; - = absent* | | | | | | | | | | | |
